# Development, Characterization and Experimental Validation of a Cultivated Sunflower (*Helianthus annuus* L.) Gene Expression Oligonucleotide Microarray

**DOI:** 10.1371/journal.pone.0045899

**Published:** 2012-10-26

**Authors:** Paula Fernandez, Marcelo Soria, David Blesa, Julio DiRienzo, Sebastian Moschen, Maximo Rivarola, Bernardo Jose Clavijo, Sergio Gonzalez, Lucila Peluffo, Dario Príncipi, Guillermo Dosio, Luis Aguirrezabal, Francisco García-García, Ana Conesa, Esteban Hopp, Joaquín Dopazo, Ruth Amelia Heinz, Norma Paniego

**Affiliations:** 1 Instituto de Biotecnología, Centro de Investigaciones en Ciencias Agronómicas y Veterinarias, Instituto Nacional de Tecnología Agropecuaria, Hurlingham, Buenos Aires, Argentina; 2 Facultad de Agronomía, Universidad de Buenos Aires, Ciudad Autónoma de Buenos Aires, Argentina; 3 Departament of Bioinformatics and Genomics, Centro de Investigación Príncipe Felipe, Valencia, España; 4 Cátedra de Estadística y Biometría, Facultad de Ciencias Agropecuarias, Universidad Nacional de Córdoba, Córdoba, Argentina; 5 Laboratorio de Fisiología Vegetal, Unidad Integrada Universidad Nacional de Mar del Plata, Estación Experimental Agropecuaria INTA Balcarce, Balcarce, Buenos Aires, Argentina; 6 Consejo Nacional de Investigaciones Científicas y Técnicas, Ciudad Autónoma de Buenos Aires, Argentina; 7 Facultad de Ingeniería, Universidad de Buenos Aires, Ciudad Autónoma de Buenos Aires, Argentina; 8 Facultad de Ciencias Exactas y Naturales, Universidad de Buenos Aires, Ciudad Autónoma de Buenos Aires, Argentina; 9 Escuela de Ciencia y Tecnología, Universidad Nacional de San Martín, San Martín, Buenos Aires, Argentina; 10 Functional Genomics Node, National Institute of Bioinformatics, Centro de Investigación Príncipe Felipe, Valencia, Spain; University of North Carolina at Charlotte, United States of America

## Abstract

Oligonucleotide-based microarrays with accurate gene coverage represent a key strategy for transcriptional studies in orphan species such as sunflower, *H. annuus L.*, which lacks full genome sequences. The goal of this study was the development and functional annotation of a comprehensive sunflower unigene collection and the design and validation of a custom sunflower oligonucleotide-based microarray. A large scale EST (>130,000 ESTs) curation, assembly and sequence annotation was performed using Blast2GO (www.blast2go.de). The EST assembly comprises 41,013 putative transcripts (12,924 contigs and 28,089 singletons). The resulting Sunflower Unigen Resource (SUR version 1.0) was used to design an oligonucleotide-based Agilent microarray for cultivated sunflower. This microarray includes a total of 42,326 features: 1,417 Agilent controls, 74 control probes for sunflower replicated 10 times (740 controls) and 40,169 different non-control probes. Microarray performance was validated using a model experiment examining the induction of senescence by water deficit. Pre-processing and differential expression analysis of Agilent microarrays was performed using the Bioconductor limma package. The analyses based on p-values calculated by eBayes (p<0.01) allowed the detection of 558 differentially expressed genes between water stress and control conditions; from these, ten genes were further validated by qPCR. Over-represented ontologies were identified using FatiScan in the Babelomics suite. This work generated a curated and trustable sunflower unigene collection, and a custom, validated sunflower oligonucleotide-based microarray using Agilent technology. Both the curated unigene collection and the validated oligonucleotide microarray provide key resources for sunflower genome analysis, transcriptional studies, and molecular breeding for crop improvement.

## Introduction

Sunflower (*Helianthus annuus* L.) is an important source of edible oil and its uses have expanded to include new markets like biofuels, biolubricants and biopharmaceuticals [1]. Sunflower breeding for agronomic trait improvement, including yield, resistance to herbicide, abiotic and biotic stresses, has contributed to yield maintenance counteracting the competition for favorable agro-ecological environments imposed during the last 10 years by increasing soybean and maize production. Advances in sunflower genomics since 1995 have greatly enhanced the development and application of new tools for crop improvement [2], [3], [4], and promoted the expansion of sunflower uses. However, sunflower genome sequencing was not approached until the advent of next-generation sequencing technologies [5] and is still in progress. In this context, providing new insights into the sunflower genome is essential to enable efficient transcriptome analyses and molecular breeding. For transcriptional studies, during the last decade, cDNA macro and microarrays were developed to study cultivated sunflower seed development [6], and the responses to biotic [7], and abiotic stresses [8], [9], [10]. Arrays based on cDNAs were also developed to carry out studies in wild *Helianthus* [11], [12] including hybrid species [13]. This approach although useful is confined to the analysis of a limited set of genes. Currently, the shortage of candidate genes underlying agronomically important traits represents one of the main drawbacks in sunflower molecular breeding. In this context, functional tools such as a high-density oligonucleotide microarray would enable the discovery and characterization of important novel genes affecting key agronomic traits. Oligonucleotide-based chips are considered more accurate than cDNA-based chips because they require fewer manipulation steps, ensuring higher reproducibility [14]. The possibility of implementing this technology in a custom array system like Agilent, NimbleGen, or others, has the potential to create a highly useful tool for gene discovery in non-model crops [15], [16]. Clearly, most plant species do not have microarrays available for gene expression analysis, although a number of attempts are being made, mainly for plants with unsequenced genomes [15]. Condensed gene indexes based on small and large scale assemblies were successfully used in model plants for the development of microarray analysis, e.g., *Arabidopsis*, tobacco, melon and rice; [17], [18], [19], [20] and non-model economically relevant plants such as maize, tomato, cotton, citrus, cucumber, Brassica, wheat, flax and coffee [21], [22], [23], [24], [25], .

Recently, both Affymetrix and NimbleGen technologies have been applied to the development of chips for the *Helianthus* genus and weeds of the Compositae family, respectively [30], [31]. The Affymetrix GeneChip was designed based on wild and cultivated sunflower raw ESTs available in the public databases [31]. The NimbleGen platform comprises one 4-plex microarray developed from the assembly of Sanger ESTs from several *H. annuus L.* cultivars deposited in GenBank up to the year 2007, and one 12-plex array based on the 454 Titanium platform transcriptome assembly from one weedy *H. annuus L.* genotype [30]. Using the same public Helianthus EST data set, plus 454 sequences from the HA89 inbred line transcriptome, a *Helianthus* gene reference assembly was built to conduct SNP discovery and to design an Illumina Infinium BeadChip for genotyping [32]. However, the use of a longer probe format represents an advantage of the Agilent oligonucleotide microarrays over other technologies, because the longer oligonucleotides provide a higher hybridization stability for sequence mismatches; consequently, the longer oligonucleotides are more suitable for the analysis of highly polymorphic regions [33].

In this work, we present the development of a comprehensive Sunflower Unigene Resource, its functional annotation and the design and validation of a custom sunflower oligonucleotide-based microarray for identification of key regulatory genes for molecular breeding and examination of concerted transcriptional responses such as those associated with leaf senescence. This development represents an initiative of the Sunflower Argentinean Consortium, working in collaboration with the Prince Felipe Research Center, Valencia, Spain, within the frame of a public research project.

## Results

### Assembly and annotation of sunflower unigenes

In this study, public and proprietary *H. annuus L.* EST datasets were used to create a comprehensive unigene collection. These datasets include ESTs from cDNA libraries described at NCBI (http://www.ncbi.nlm.nih.gov/UniGene/lbrowse2.cgi?TAXID=4232&CUTOFF=100) up to February 2009. These ESTs were derived from different cultivated sunflower lines and cultivars, from various tissues and anatomical parts, and from plants grown under different physiological conditions. After cleaning and removal of low quality and short (<100 bp) sequences, the dataset was reduced to 132,479 reads. Also, additional ESTs or gene sequences of special interest for relevant traits were added to the initial dataset. Clustering and assembling of 133,682 ESTs was conducted using CAP3 [34] with parameters set according to the most relevant and recently published microarray designs (p = 95, f = 45, h = 25, o = 80) [22], [24], [35], [36], [37]. The final assembly resulted in 41,013 putative transcripts (12,924 contigs and 28,089 singletons) (Figure S1). To show that the public EST collection for *H. annuus L.* was well represented and that no bias and/or enrichment for specific transcripts might have occurred [38], a digital expression analysis was conducted using the total set of ESTs available for cultivated sunflower at NCBI (http://www.ncbi.nlm.nih.gov/nucest). The digital expression analysis estimates the probability for a gene to be equally expressed in two different conditions by observing the distribution of tag counts (ESTs) for each library, showing that the sunflower EST dataset had an unbiased differential representation for each tag [39].

**Figure 1:**
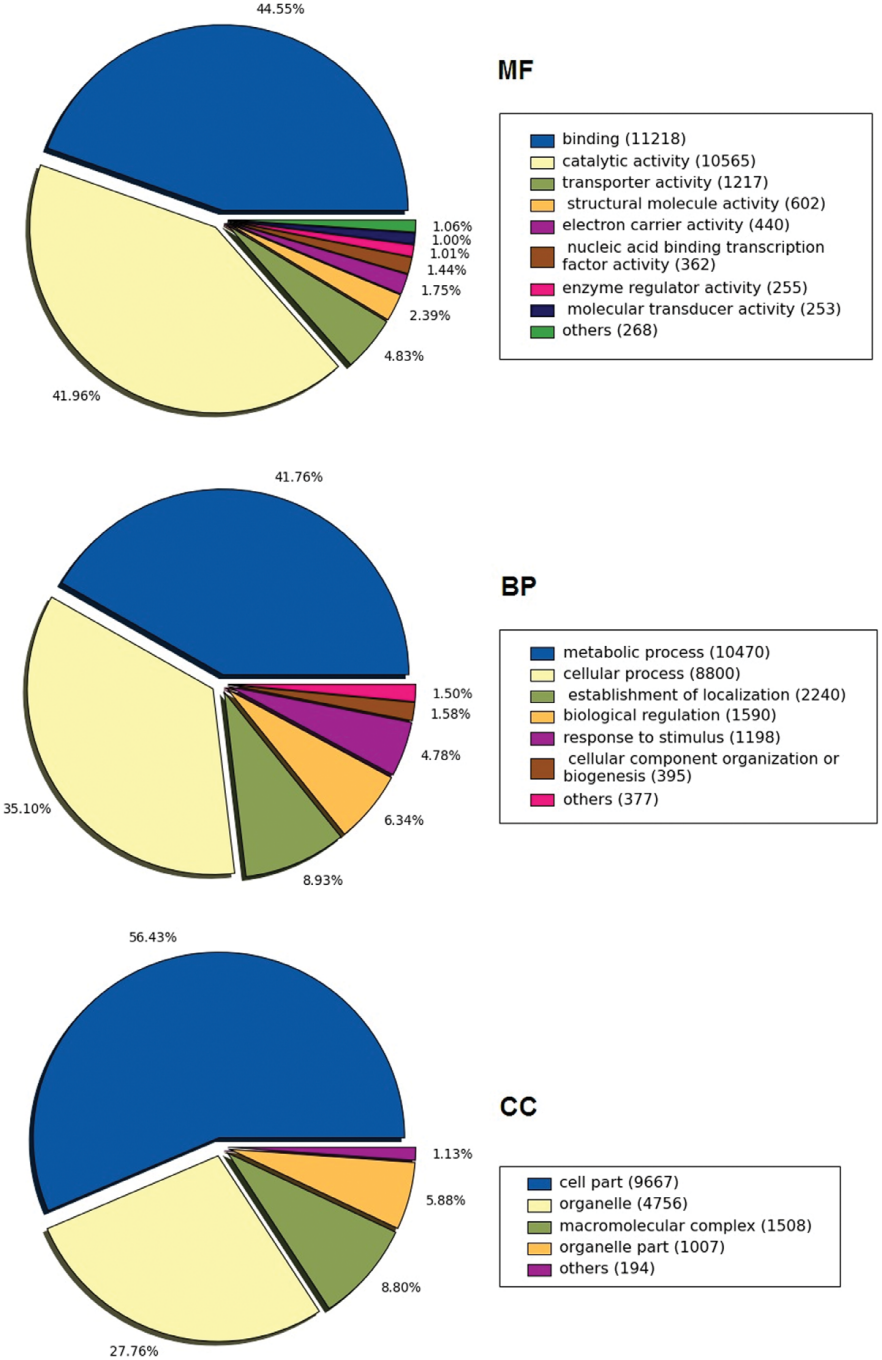
Pie charts showing predicted gene products using Gene Ontology terms. Data was obtained from SUR v 1.0.

The EST clustering and assembly resulted in a set of unigenes, the Sunflower Unigen Resource v 1.0 [40]. To further annotate the SUR, Gene Ontology annotations were assigned using the program Blast2GO [41], which implements a functional annotation strategy that takes into account sequence similarity searches, GO term evidence codes and the gene ontology structure to provide quality functional predictions [42]. InterProScan searches were also implemented during the annotation process, InterPro [43] is an integrated resource for protein families, domains, regions and sites. Annotated genes were classified according to GO categories: cellular component (CC), biological process (BP) and molecular function (MF). According to this analysis it can be inferred that SUR v 1.0 appears to be functionally diverse; for example, metabolic and cellular processes are the most-represented GO terms within the BP category, cell part and organelles are the most-represented terms in the CC category, and binding and catalytic activity are the most-represented terms in the MF category. Figure 1 depicts the distribution of the major GO categories, and more details on each category can be found in the SUR database (at http://atgc-sur.inta.gob.ar). Overall, this GO term representation is comparable to others reported for relevant species with a comprehensive annotated gene index [25], [44]. By contrast, transport, regulation, response to stimulus and biogenesis are poorly represented, probably due to the biological attributes of the tissues from which the ESTs derive (bud, embryo, flower, leaf, root, stem among other not yet classified.

### Microarray design

The SUR v 1.0 data was submitted to the Agilent eArray web tool for gene expression probe design (http://atgc-sur.inta.gob.ar). This resulted in a total of 42,386 different non-control probes, 74 sunflower control probes replicated 10 times (740 controls) [10] and 1,417 Agilent controls. Analysis of probes and unigenes showed that out of the total non-control probes, 35,879 probes were associated with a single gene, 1,989 were predicted to produce high cross-hybridization with other genes and were discarded, and 4,290 showed cross-hybridization to only a few targets and therefore were included in the array, to be filtered later when differential gene expression analysis was assessed. Considering the full annotations achieved (Table S1) there are 38,485 GO terms resulting in 49.6% of total sequences having a GO annotation. The Sunflower Unigen Resource [45] v 1.0 allowed the generation of a microarray platform which is available at the NCBI Gene Expression Omnibus (GEO) according to MIAME guidelines [46], under the Accession Number GPL13610, whereas raw data corresponding to chip validation (see below) is deposited under Accession Number GSE29390.

### Microarray validation

To confirm that the sunflower microarray can be used to generate biologically useful information, we used this platform to analyze global changes in gene expression profiles in response to water deficit, as a physiological event that induces senescence. This approach was chosen as a performance test experiment because senescence reference genes were previously identified and validated by our group [47]. Hence, the experimental model used to test the 44 K Sunflower Chip comprised field samples corresponding to leaf 15 of sunflower plants growing under control and mild water deficit conditions as described in Material and Methods. RNA was isolated for each treatment and replicates, making a total of six independent samples. Gene expression profiles were generated by labeling and hybridizing each sample to one of six separate microarrays. FatiScan [48] was used to detect blocks of genes functionally related by different criteria such as gene ontology terms.

### Response to RNA concentration

**Figure 2:**
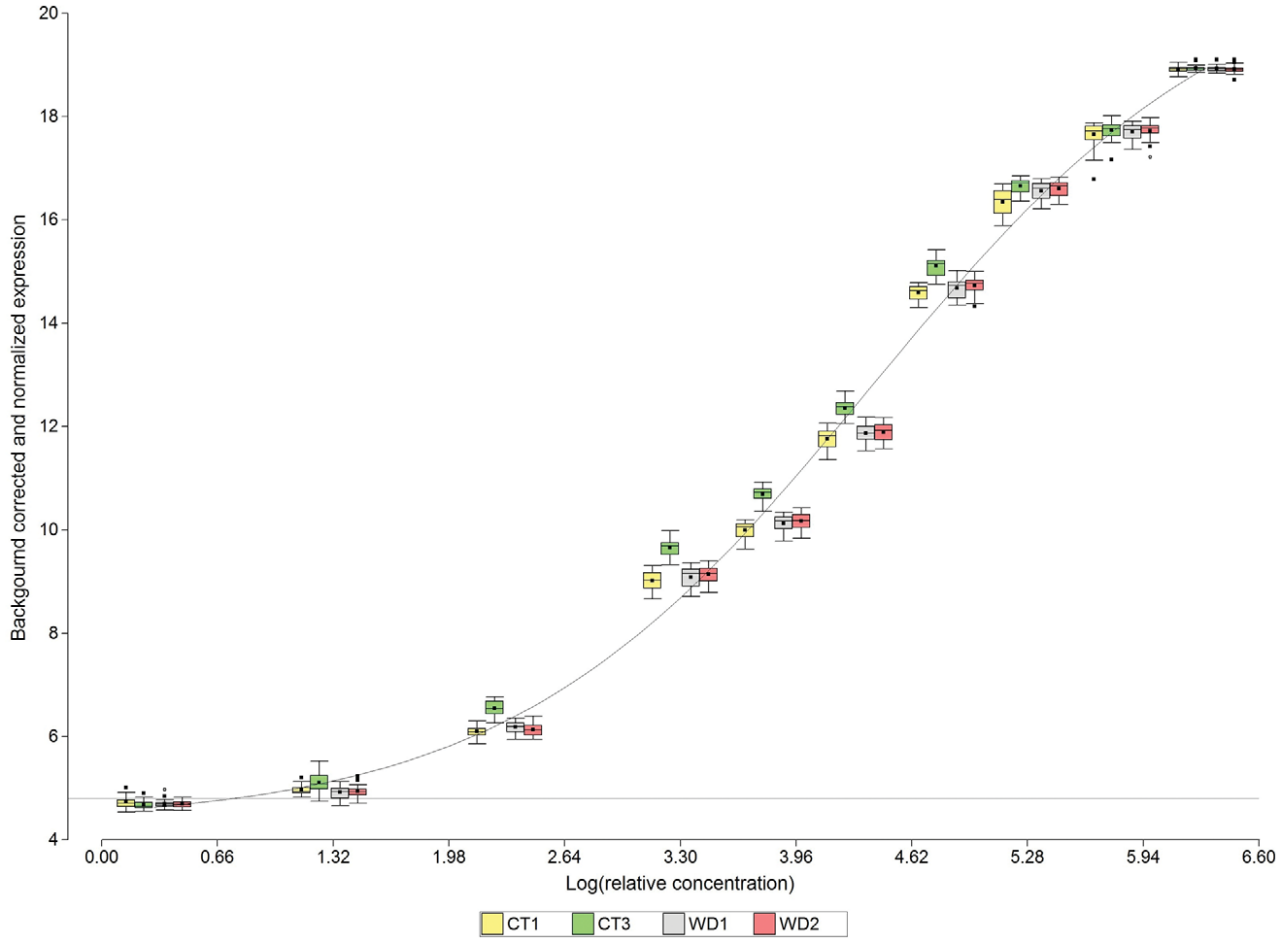
Normalized expression for RNA Spike-in control samples. The logistic curve was fitted for the average of all treatments and replicates including a baseline at 4.7 units in the Y-axis scale. This baseline corresponds to the 5% lowest percentile of the expression signal distributions for RNA-Log (relative concentration) of 1.3. Box-plots of observed gene expression signals of technical replicates within each biological replicate, at different relative RNA concentrations in log scale, are represented.

We also performed key controls to check the sensitivity of the array to RNA concentrations and to define the concentration ranges in which the array response is proportional to RNA concentration (Figure 2). Agilent microarrays include the Spike-In Kit, consisting of a set of 10 positive control transcripts optimized to anneal to complementary probes on the slide, minimizing self-hybridization or cross-hybridization. The Agilent One Color RNA Spike-Mix stock was diluted with the buffer provided (Agilent Technologies Inc., USA). Diluted RNA controls were spiked directly into the RNA samples prior to amplification and labeling to achieve the correct relative amounts of standard (www.genomics.agilent.com/files/Manual/5188-5977.pdf). Figure 2 also shows the expression signal of the Spike-in controls as a function of log relative concentration. Box-plots of observed gene expression signals of technical replicates within each biological replicate, at different relative RNA concentrations in log scale, are presented. Figure 2 includes a logistic curve describing the relationship between expression signals and log relative concentration. The curve was fitted to the average of expression signals across all treatments and their replicates. According to these results, expression signals increase according to increasing RNA concentration. Moreover, the logistic trend follows the expected shape of the relationship between readings and RNA concentration (log scale) according to the Agilent One Color RNA Spike-In Kit technical report (www.genomics.agilent.com/files/Manual/5188-5977.pdf).

### Sensitivity

**Figure 3:**
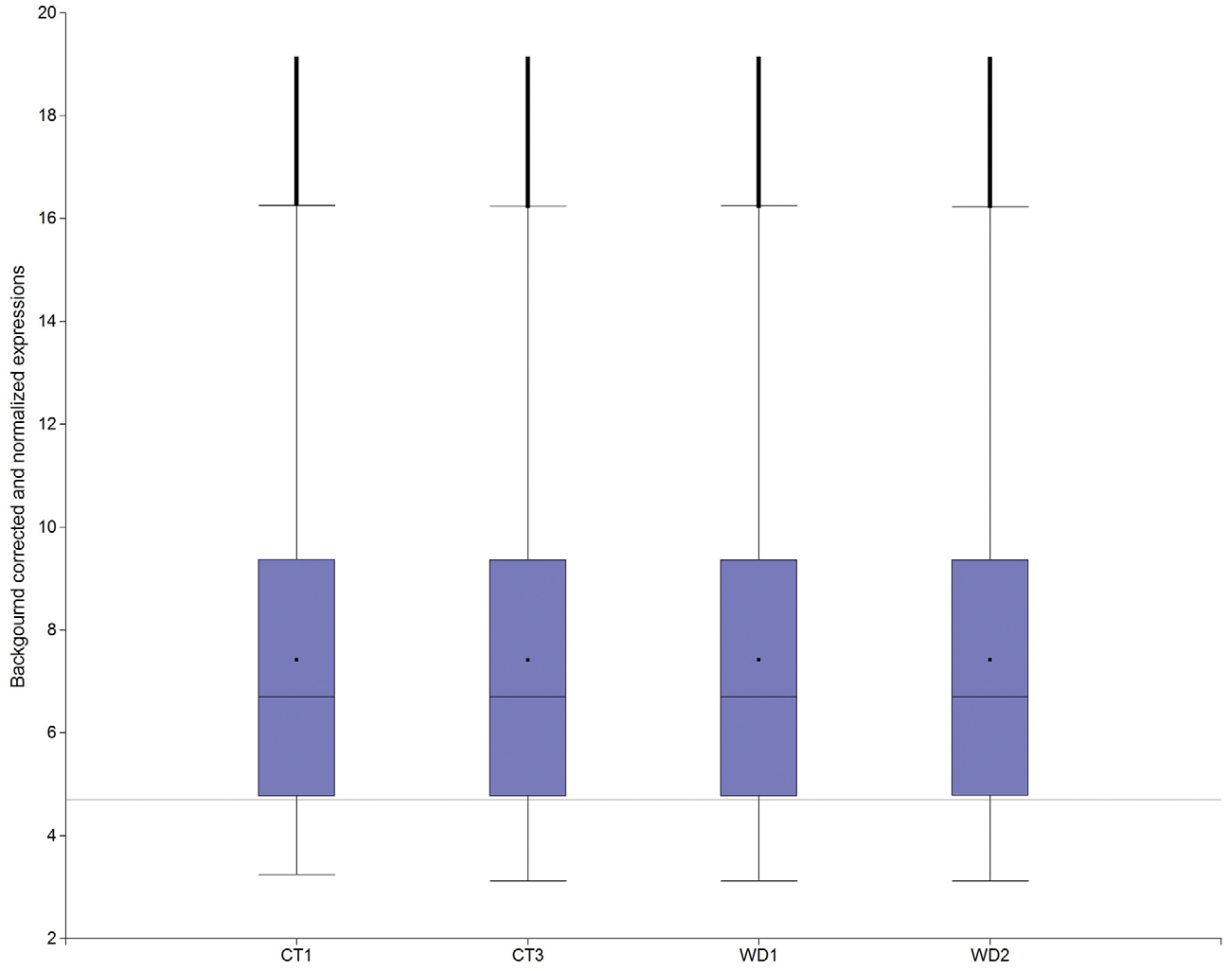
Box-plot of the normalized expression in each replicate of both treatments. Base line at 4.7 describes a sensitivity limit for detection of changes in RNA concentration.

The relationship between expression signals and relative RNA concentration (log scale) increased according to a logistic curve; therefore, at upper and lower RNA concentrations, signal expression does not reflect changes in concentration. When RNA concentrations are lower, signal is not distinguishable from the background; when RNA concentrations are higher, signal becomes saturated. Figure 2 includes a baseline at 4.7 units in the Y-axis scale. This baseline corresponds to the 5% lower percentile of the distribution of expression signal for an RNA-Log (relative concentration) of 1.3. Expression levels lower than this threshold value should be considered background for this assay. Under this assumption, this sensitivity cut-off value explains why almost 20% of the expression readings in the set of genes are considered background noise (Figure 3).

### Variability of expression signals at different RNA concentrations

At intermediate RNA concentrations, the variability of the expression signal is greater than the variability observed at low and high RNA concentrations. This result differs from Agilent's reports, which describe greater variability in the lower end of the concentration range. However, we consider that a greater variability in the intermediate concentrations is consistent with the fact that a sigmoid curve has low and high asymptotes, which in turn implies that gene expression signals are bounded at low and high concentrations. Coefficients of variance (CV%), for technical replicates are shown in Table 1. The CVs look low (ranging from 0.33 to 3.85%) compared to data provided by the Agilent One Color RNA Spike-In Kit technical report, where CVs range from near 5% to more than 100%. Even taking into account variability among biological replicates, CVs remain below 5% (Table 1).

**Table 1:** CV estimation for technical and biological replicates.

Log(RC)	CT1	CT3	WD1	WD2	CT	WD
**0.30**	2.51	2.04	1.65	1.57	2.34	1.60
**1.30**	1.82	3.85	2.46	2.34	3.29	2.39
**2.30**	1.86	2.17	1.83	1.96	4.09	1.93
**3.30**	1.83	1.55	2.05	1.57	3.84	1.84
**3.83**	1.56	1.33	1.53	1.57	3.62	1.55
**4.30**	1.58	1.27	1.45	1.44	2.86	1.44
**4.82**	1.02	1.14	1.20	1.16	2.05	1.19
**5.30**	1.55	0.89	1.11	1.01	1.57	1.06
**5.82**	1.29	0.93	0.79	0.89	1.14	0.84
**6.30**	0.34	0.33	0.33	0.45	0.34	0.39

RC: relative concentration.

CT: control.

WD: water deficit.

Technical variability within biological replicates (columns: CT1, CT3, WD1, WD2); technical variability plus biological variability within each treatment (Columns: CT and WD).

### Differential gene expression

Genes having differential expression between treatments were identified using *the lmFit* and *contrasts.fit* functions of the limma Bioconductor package [49]. Due to the reduced number of replicates and treatments, there were few degrees of freedom for the estimation of experimental error variances. Thus, an empirical Bayes procedure (eBayes) was used to improve the estimation and augment the degrees of freedom for the individual variances. Differentially expressed genes were identified according to their p-values after eBayes correction using a significance level of 0.01. In total, 558 genes differentially expressed between control and water deficit conditions were identified (Table S2).

As already indicated, the microarray chip contains control and Agilent probes (including spike-in), which are not expected to be affected by treatments. The microarray chip also contains “non-control” probes; a subset of these will show alterations in signal in response to the treatments. Within the “non-control” group there is a positive control subset called “sunflower control”, which includes 79 genes previously recognized as sensitive to abiotic stress [10]. In the Agilent controls, only 1.7% of probes were identified as differentially expressed, in the non-control genes 8% were differentially expressed, and in the sunflower control probe set, 18% were identified as differentially expressed. These results are consistent with the results expected from controls and non-control genes included in the oligo-based chip.

Gene set analysis was carried out using Gene Ontology terms (GO terms) [50] by running FatiScan [48], which is integrated in the Babelomics suite [51]. This method detects significantly up- or down-regulated clusters of functionally related genes in lists ordered by differential expression. Therefore, FatiScan examined functional differential expression by looking for over-represented ontologies in the Babelomics ranked gene list. As expected, considering the abiotic stress treatment used for chip validation, water and anion transport categories were enriched. However, responses to abscisic acid stimulus, salt and cold acclimation categories were under-represented (Figure S2). Major biochemical pathways of sucrose, fatty acid and carbohydrate biosynthesis, as well as tyrosine, tryptophan, L-serine, histidine and glycine biosynthetic pathways, were also overrepresented in water deficit conditions. Functional categories including response to stress mechanisms, comprising signal transduction and regulation of salicylic acid metabolism, were also overrepresented, among others.

### qPCR analysis for differentially expressed genes

To validate candidate genes identified from the microarray analysis, quantitative real time PCR (qPCR) was performed on the same mRNA samples used for the microarray experiments. Ten genes were selected for qPCR analysis from the 558 differentially expressed genes, based on e-Bayes corrected p-value rankings and Fatiscan output results based on relevant functional GO categories (Table S2). The expression profiles of these genes were estimated in relation to reference genes using fgStatistic software, which uses previously published algorithms [52]. These results showed statistically significant differences (p-value≤0.05) for nine of ten genes assessed, with a ratio of expression consistent with the statistical analysis of the microarray results (Table 2 and Table S2).

**Table 2:** qPCR results for ten selected differentially expressed genes.

Gene	Sequence 5′-3′	*R^2^*	Efficiency (%)	Amplicon size pb	Ratio Stress vs Control	p-value
HeAn_C_266	Forward CCATCGAACTAAGGCCACATReverse CACGCAAAGCTCCAACATAA	0.999	91	166	7.05	0.0326
HeAn_C_3312	Forward TTCTTCCCCACCCTTTTTCTReverse GAGGTTCTGATCGGTGTGGT	1.000	88	143	2.04	0.0518
HeAn_C_5545	Forward CCGGAAATCGTTGTTCAAGTReverse TCAGTGACACGAACGAGACC	0.998	93	164	4.79	0.0344
HeAn_C_2759	Forward CCCGAGTTGCAAAAAGTTGTReverse CCCTTCATTTGCATTGCTTT	0.999	87	135	2.67	0.0236
HeAn_C_9326	Forward AACCCAAGTTTGATCCATGCReverse GGTCAGCCACCTCACGTAAT	0.993	100	119	1.12	0.3721
HeAn_C_593	Forward GCGACAGAAGAAAAGGCAACReverse TGCAACCAGATCTGAAGACG	1.000	88	167	3.38	0.0412
HeAn_C_1482	Forward AGCCGTTACATCCCCTCTTTReverse ACAACCCGGGGATTCTACTC	0.999	87	122	4.7	0.043
HeAn_C_4439	Forward GGAAACATAGGTTGCGAGGAReverse CCTTTGACCCGTCTTTTCAA	1.000	86	101	3.51	0.0292
HeAn_C_2975	Forward ATCGACATCCCACACAGTGAReverse AACATGCCCACCGTAAATGT	0.997	96	102	2.10	0.0331
HeAn_C_4809	Forward GACGTTGAACGGGTCTTGTTReverse TGAAGCAACGCCTGATTATG	0.999	88	164	2.82	0.0222

## Discussion

Microarray technology first opened a new era of high-throughput transcriptome analysis approximately fifteen years ago [53], [54]. Although next-generation sequencing technologies can explore and analyze transcriptomes from large genomes, for many species the lack of a reference genome provides a major constraint to extracting significant biological information. Because of this constraint, non-model species are excellent targets for genome studies using different strategies for gene-index construction [55]; these studies thus contribute concise transcriptomic data to improve our biological understanding of diverse processes [56]. In this context, improving coverage by accurate microarray design seems to be the most desirable application for these technologies [57]. In the particular case of sunflower, even though genome sequencing is in progress [5], there is not yet a reference genome available. Recently, two platforms for high throughput expression studies of the Compositae family have been developed based on proprietary designs [30], [31]. The Affymetrix Sunflower Array includes genes from the genus *Helianthus* and has been applied to study seed dormancy regulation in cultivated sunflower [31]. The NimbleGen Compositae Microarray Platform, based on unigenes derived from *Ambrosia artemisiifolia*, *Centaurea diffusa*, *Centaurea solstitialis*, *Cirsium arvense*, *Helianthus sp.* and *H. annuus L.*, has been developed as a genomic tool and resource for population and comparative genomic analyses within the Compositae family [30].

In the case of cultivated sunflower (*H. annuus L.*), about 133,000 ESTs are publicly available (http://ncbi.nlm.nih.gov/dbEST/dbEST_summary.html), but it is worth noting that these databases tend to be significantly contaminated with vector sequences and chimeras. These ESTs also have relatively low quality DNA information because of the library sequencing strategy, which prioritizes obtaining a large number of single pass sequences, thus leading to a concomitant decrease in the quality of the deposited sequences [58]. In consideration of this particular situation, in this study, a 60-mer oligonucleotide microarray was successfully developed for cultivated sunflower using a curated unigene database produced by the Argentinean Sunflower Consortium, which is made up of six laboratories and one private company working in different areas of sunflower research and development. The *H. annuus L.* 44 K Agilent oligonucleotide microarray was developed based on a unigene set of 28,089 singletons and 12,924 contigs obtained using the CAP3 parameters described above. These parameters, and the diverse sunflower lines used for EST generation, result in a unigene set that has strong representation of the genetic diversity due to gene duplication, allelic variation and other factors. Additional sequence variation is due to sequencing errors present in the different EST libraries. Fortunately, the Agilent 60-nt oligonucleotide arrays should be robust to such variation, providing reliable readout of expression data. Biological interpretation of data generated by microarray analysis for non-model species will also be enriched substantially by further validation experiments, which will improve our poor understanding of plant genomes and mitigate the low quantity of GO terms defined for these organisms [59]. Considering that whole sequences were selected from public web deposited ESTs, which are based on non-normalized, normalized and SSH cDNA libraries from different developmental stages and tissues, and taking into consideration that sunflower is a non-model crop with no genome sequence available, this microarray represents a key tool offering high coverage of genes involved in diverse biochemical pathways, according to the metabolic annotations we conducted on the SUR v. 1.0 unigene set. Moreover, GO term mapping was carefully done, using Blast2GO [41] against a local GO database (2011-08 update). Annotation was completed by running a local installation of InterProScan v4.7 follow by InterPro2GO (database version 31.0, release February 2011); for that analysis, we considered whole sequences with BLASTX hits and used the same reading frame, but for anonymous sequences we considered 6-frame translations. To store, visualize, analyze and share this information, plus the probes associated to each unigene represented in the microarray, we created a unigene collection database available at http://atgc-sur.inta.gob.ar. Indeed, numerous transcripts, variants and new genes will be identified as candidate genes for relevant biological processes in sunflower, making an enormous contribution to the Compositae research community.

The validation of the microarray for analysis of transcriptional profiles was performed using sunflower leaves derived from plants grown under two conditions, including biological and technical replicates. The degree of sensitivity in response to RNA concentration and the variability among replicas for the Agilent Sunflower Microarray were satisfactory.

An alternative approach to extract biologically relevant information from genome-wide microarray analysis is to use threshold-free functional enrichment methods such as Gene Set Enrichment Analysis (GSEA) [60]. In GSEA, genes are not selected on the basis of a hypothesis-driven analysis, but rather the derived statistics are used to rank genes according to their association to the phenotype and groups of functionally related genes with similar expression changes are sought along the list of ranked genes. In our study, the GO processes “cellular wall”, “protein complex assembly” and “response to stress” were the most represented functions, in agreement with the biological activities expected to be overrepresented. Moreover, microarrays for other non-model plant species show a similar distribution of GO BP processes, especially for “response to stimulus” and “cellular process” GO categories. However, as GO terms are dynamically updated, many terms mapped for the SUR v1.0 collection could differ from other annotations reported in other differential gene expression studies [21], [22], [23], [24], [26], [61], [62]. Our results show that the *H. annuus L.* microarray is suitable for functional genomics analysis. Indeed, this microarray is already being used for other experiments and shows precise and accurate results with a high level of trustability for different gene expression profiles.

We cannot make a precise comparison of the gene representation in the SUR microarray to gene representation in the recently described *Helianthus sp* and Compositae microarrays because we do not have access to the precise array designs. However, we can infer this information by considering that the three platforms were developed with shared raw data from *Helianthus* EST public sources. For example, the SUR array and the Sunflower Affymetrix GeneChip share the whole set of raw ESTs from *H. annuus L.*, although the design of the SUR array was based on a locally curated gene index. Also, the Sunflower Affymetrix GeneChip probes represent wild and cultivated sunflower ESTs. The SUR array and the 4-plex *H. annuus* NimbleGen array share some of the raw EST data from *H. annuus L.*, since the NimbleGen array used the Sanger EST libraries for *H. annuus L.*, as previously described [63] and as available at the website of the Compositae Genome Project DB [30]. This dataset lacks 39,702 ESTs generated from the HA89 inbred line and released by the Compositae Genome Project by the end of 2008. Considering these differences, the SUR array indeed complements the 4 plex *H. annuus L.* NimbleGen microarray [30] and the *Helianthus* genus Affymetrix microarray [31] resources, representing a specific collection of probes useful for interrogating expression profiles in sunflower crops under different physiological conditions. The use of a microarray that specifically represents the *H. annuus L.* transcriptome (including allelic variants), can improve the analysis of data and interpretation of results from cultivated sunflower experiments. This approach is highly recommended, especially considering that most sunflower transcriptional studies, especially those involving vegetative or reproductive stages, must be carried out in field conditions. However, we note that the *Helianthus* genus array and the 12-plex *H annuus L.* NimbleGen platform are more suitable for population, comparative and evolutionary studies, but less suitable for crop specific studies.

In the future, this transcriptome tool will be added to the full sunflower genome sequence data, which is currently in process [5], and will facilitate comparative and functional analysis of *Asteracea*, one of the most diverse families of flowering plants. The *Asteracea* are also agronomically relevant, with very little available genomic information. This work generated a curated and trustable sunflower unigene collection, which resulted in a custom sunflower oligonucleotide-based microarray using Agilent technology. The work presented here gives the cultivated sunflower research community a trustable microarray to use for different transcriptional profiling applications.

## Materials and Methods

### EST assembly and annotation

A total of 133,682 EST sequences of *H. annuus L.* were downloaded from Genbank (http://www.ncbi.nlm.nih.gov/nucest) in December 2008. These sequences were screened for the presence of remnants of cloning or sequencing vectors by running BLASTN [64] optimized for short matches against the UniVec database (ftp://ftp.ncbi.nih.gov/pub/UniVec/). Any contaminating sequence located at either end of the ESTs was trimmed. Those ESTs containing contaminating vector sequence in the middle region were discarded. Thus, a total of 1,162 ESTs were removed. Additionally, 41 ESTs whose length was shorter than 40 bases after trimming were discarded. ESTs contained regions with high frequencies of ambiguities (N's) on one or both ends were trimmed using the program TrimSeq from the EMBOSS suite [65]. Nine percent of the ESTs, containing poly-A tails on the 3′ end or poly-T on the 5′ end, were clipped using Trimest from the EMBOSS suite.

As many members of this assembly lacked sense orientation, BLASTX analysis was run using the CAP3 assembly against the protein RefSeq database (http://www.ncbi.nlm.nih.gov/RefSeq/) in order to choose the best hit for every sequence and to infer the correct orientation of the unigenes. An additional subset of 120 new EST sequences derived from local SSH-identified transcripts that had not been deposited in Genbank at the time of downloading was compiled into the former assembly.

Sequence annotation was performed using different tools. First, we ran 12,924 contigs and 28,089 singletons against all public plant protein sequences available at GenBank (January 2011), using BLASTX (E<10^−10^) [64]. As a result, we obtained 25,988 sequences with BLASTX hits. These results were fed into Blast2GO under default parameters [41], using a local database generated from the GO database (2011-08 update), idmapping.tb dated 2011-07-27, gene_info and gene2accesssion downloaded on 2011-08-18. In addition, all translated amino acid sequences with BLASTX hits, as well as sequences lacking BLASTX hits, were previously translated into 6 frames, and were fed into InterProScan (http://www.ebi.ac.uk/Tools/pfa/iprscan/) so as to annotate them. We used a local installation of InterProScan v4.7 (database version 31.0, release February 2011). Further GO annotation was performed by mapping InterProScan results to GO using the InterPro2GO tables. Using both approaches, we obtained 38,485 GO annotations for 8,369 contigs and 48,830 for 11,983 singletons. This annotation procedure resulted in 49.6% of total sequences with GO annotation.

### Microarray design and synthesis

For the custom Gene Expression chip design, the Agilent Technologies eArray® web application was used. Probe sequences were obtained using GE Probe Design considering 3′ end biased 60mer oligonucleotide, one probe per target, vector sequence and masking function on probe sense orientation. Two probe sets were designed: one including non-control specific probes for the Sunflower Unigene Resource (SUR v 1.0) and a sunflower control probe set consisting of 74 probes (10 times replicated) derived from 80 differentially expressed sunflower genes identified in a previously work [10]. To utilize the full capacity of the microarray, probes were randomly selected to be represented in duplicate in the final design, which also included Agilent Technologies' standard panel of quality control and spike-in probes. This design was then used to manufacture microarrays using Agilent SurePrint™ Technology in the 4×44 format. Agilent's microarrays include the Spike-In Kit that consists of a set of 10 positive control transcripts optimized to anneal to complementary probes on the microarray, minimizing self-hybridization or cross-hybridization. The concentrated Agilent One Color RNA Spike-Mix stock was diluted with buffer provided with the kit. The diluted RNA controls were spiked directly into the RNA samples, prior to amplification and labeling, to achieve the correct relative amounts (www.genomics.agilent.com/files/Manual/5188-5977.pdf).

### RNA isolation and quality controls

Total RNA isolation was performed on healthy green leaf samples from 48-day-old plants in order to assure RNA integrity. Samples were immediately frozen in liquid nitrogen and stored at −80°C until processing. High quality total RNA was isolated from 100 mg of frozen tissue using Trizol® following the manufacturer's instructions (Invitrogen, Argentina). Genomic DNA was eliminated by treatment with DNAse I for 20 min at RT using DNAse I® (Invitrogen, Argentina).

RNA concentration was measured using a Nanodrop ND-1000 spectrophotometer (NanoDrop Technologies, Wilmington, Delaware USA). Purity and integrity of total RNA was determined by 260/280 nm ratio and the integrity was checked by electrophoresis in 1% agarose gel and quality confirmed by RNA 6000 Nano Bioanalyzer (Agilent Technologies, Palo Alto, California USA) assay.

### cRNA synthesis, labeling and microarray hybridization

200 ng of total RNA was used to produce Cyanine 3-CTP-labeled cRNA using the Low Input Quick Amp Labelling Kit, One-Color (Agilent Technologies) according to the manufacturer's instructions. Following ‘One-Color Microarray-Based Gene Expression Analysis’ protocol version 6.0 (Agilent Technologies), 2 µg of labeled cRNA was hybridized with a Sunflower Custom Oligo Microarray (Agilent Technologies) containing 42,326 probes derived from SUR v 1.0.

Agilent's recommended protocol for microarray workflow quality control was implemented using the Agilent Spike-In Kit. This kit consists of a set of 10 positive control transcripts optimized to anneal to complementary probes on the microarray with minimal self-hybridization or cross-hybridization. The concentrated Agilent One Color RNA Spike-Mix stock was diluted in the buffer provided by the kit and mixed with the RNA samples prior to the amplification and labeling process to achieve the relative amounts recommended by the manufacturer (www.genomics.agilent.com/files/Manual/5188-5977.pdf).

### Microarray scanning and data analysis

Slides were scanned in an Agilent Microarray Scanner (G2565BA) according to the manufacturer's protocol. Signal data were collected with dedicated Agilent Feature Extraction Software (v9.5.1) following the Agilent protocol GE1_107_Sep 09 and the QC Metric Set GE1_QCMT_Sep09.

Agilent Processed Signals (generated by the Agilent Feature Extraction software) were pre-processing with functions implemented in limma package [49], available from the open source Bioconductor platform (http://www.bioconductor.org/). The background correction was done with the backgroundCorrect () function, using normexp method and offset = 1 to avoid negative values after log-transformation. Normalization was achieved using the normalizeBetweenArrays () function, applying the quantile method [66]. The raw data are available from the GEO repository, accession number GSE29390.

Differential gene expression analysis was also carried out using the limma package. Gene set analysis was carried out according to the Gene Ontology terms using FatiScan [48] integrated in the Babelomics suite [51].

### Plant materials and experimental conditions used for chip validation

A field experiment was carried out at INTA Balcarce Experimental Station (37°45′ S, 58°18′ W) during the 2004/05 growing season. Sunflower hybrid VDH 481 (Advanta Seeds) was sown on November 18^th^ at a 7.2 plants/m^2^ density. Emergence occurred 9 days later. Diseases, weeds and insects were adequately controlled. Soil fertility assured maximum yields under non-limiting water conditions. Rainfall was complemented with irrigation when necessary to avoid water deficit in control plants. Soil volumetric humidity was measured periodically using time domain reflectometry equipment (Trase System, Model 6050X1, Soil moisture Equipment Corp., Santa Barbara, CA, USA). Leaf 15 was considered to have appeared when the primordium was visible (about 40 µm long) on the apical meristem, observed under a stereomicroscope using 80× magnification (Olympus SZX12). A mild water-deficit (WD) treatment was applied to accelerate senescence in comparison to control plants. WD was achieved by covering the soil with a 200 µm plastic mesh to prevent rainfall penetration into the soil. The mesh was installed 7 days before flowering, and reduced by about 40% the volumetric humidity at a soil depth of 0.60 m. Controlled irrigation maintained the water deficit up to the sample harvest day. Senescence symptoms in water deficit leaf 15, measured as the reduction in chlorophyll content, were achieved 4 days before symptoms appeared in control plants, confirming the mildness of the water deficit (data not shown).

The experiment was conducted as a randomized complete block design with three replicates (plant-plots). Each experimental unit was integrated by three randomly selected plants from each plot.

Leaf 15 (numbered from the bottom to the top of the plant) was sampled at 48 days old from its appearance on the apical meristem, and three biological replicates were used for each treatment, control and water-deficit.

### Quantitative RT-PCR analysis

Ten genes were selected according to their highest level of expression in the water deficit treatment compared to the control condition for further qPCR validation (Table 2). Specific primers for qPCR were designed from each target sequence using Primer 3 [67] with default parameters.

For each sample, 500 ng DNAse treated RNA was reverse-transcribed using SuperscriptIII first strand synthesis system (Invitrogen, USA) and random hexamers according to manufacturer's instructions (http://tools.invitrogen.com/content/sfs/manuals/superscriptIIIfirststrand_pps.pdf). qPCR was carried out in a 25-µl reaction mix containing 200 nM of each primer, 1 µl of cDNA sample and FastStart Universal SYBR Green Master (Roche Applied Science). Negative RT RNA control and non-template controls were incorporated in the assays. qPCRs were performed using a 96-well plate thermocycler (ABI Prism 7000 Sequence Detection System and software, PE Applied Biosystems, USA). The thermal profile was set to 95°C for 10 min, and 40 cycles of 95°C for 15 s, and hybridization temperature for 1 min. Amplicon specificity was verified by melting curve analysis (60 to 95°C) after 40 PCR cycles. The qPCR assay was carried out using two biological replicates for each treatment and two technical replicates for each biological replicate, derived from independent cDNA synthesis.

Two reference genes previously characterized in sunflowers, EF-1α and α-TUB, were used as endogenous controls for expression level [47]. Amplification efficiencies and Ct values were determined for each gene and each tested condition, with the slope of a linear regression model using the *LinRegPCR* [68]. Analyses of quantitative RT-PCR data are listed in Table 2. The expression profiles of these genes were estimated in relation to reference genes using *fgStatistic* software [69], which uses previously published algorithms [52].

## Supporting Information

Figure S1 **Flow chart including curation, assembly and annotation routines applied to construct SUR v 1.0.** Information derived from public sunflower ESTs (*H. annuus L.*).
(TIF)

Figure S2 **Significant GO BP for differentially expressed genes in WD.**
(TIF)

Table S1 **GO terms for SUR v 1.0.**
(XLSX)

Table S2 **Differentially expressed genes in WD against CT (adjusted p-value<0.05).** Genes ID, statistical p-values and adjusted p-values are included for all genes on the microarray. Over-expressed genes in the first condition are listed first and the most repressed genes are at the bottom of the list.
(XLS)
