# Supplementary figures and images for: Development, Characterization and Experimental Validation of a Cultivated Sunflower (Helianthus annuus L.) Gene Expression Oligonucleotide Microarray
Source: PLoS One. 2012 Oct 26;7(10):e45899. doi: 10.1371/journal.pone.0045899 (PMC3482228; doi:10.1371/journal.pone.0045899)

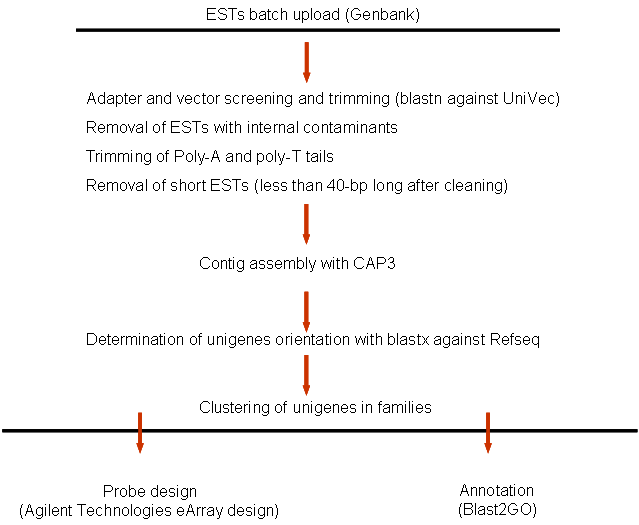

Supplement: Figure S1 — Flow chart including curation, assembly and annotation routines applied to construct SUR v 1.0. Information derived from public sunflower ESTs (H. annuus L.). (TIF) [file pone.0045899.s001.tif]
